# *ERBB3*-rs2292239 as primary type 1 diabetes association locus among non-*HLA* genes in Chinese

**DOI:** 10.1016/j.mgene.2016.05.003

**Published:** 2016-05-12

**Authors:** Chengjun Sun, Haiyan Wei, Xiuli Chen, Zhuhui Zhao, Hongwei Du, Wenhui Song, Yu Yang, Miaoying Zhang, Wei Lu, Zhou Pei, Li Xi, Jian Yan, Dijing Zhi, Ruoqian Cheng, Feihong Luo

**Affiliations:** a Department of Pediatric Endocrinology and Inherited Metabolic Diseases, Children's Hospital of Fudan University, Shanghai, China; b Department of Pediatric Endocrinology, Children's Hospital of Zhengzhou, Zhengzhou, Henan, China; c Department of Pediatric Endocrinology, Soochow University Affiliated Children's Hospital, SuZhou, Jiangsu, China; d Department of Pediatric Endocrinology, First Affiliated Hospital of Jilin University, Changchun, Jilin, China; e Department of Pediatric Endocrinology, Children's Hospital of Shanxi Province, Taiyuan, Shanxi, China; f Department of Pediatric Endocrinology, Children's Hospital of Jiangxi Province, Nanchang, Jiangxi, China; g Science for Life Center, Department of Biosciences and Nutrition, Karolinska Institutet, Huddinge, Stockholm, Sweden

**Keywords:** Type 1 diabetes, Genetic association, Single nucleotide polymorphism, ERBB3, T1D, type 1 diabetes, GWAS, genome-wide association study, HLA, human leukocyte antigen, SNP, single nucleotide polymorphism, DNA, deoxyribonucleic acid, SBE, single-base extension, HWE, Hardy-Weinberg equilibrium, OR, odds ratio, CI, confidence interval, EGFR, epidermal growth factor receptor

## Abstract

Type 1 diabetes (T1D) is an autoimmune disease that has strong contribution of genetic factors to its etiology. We aimed to assess the genetic association between non-*HLA* genes and T1D in a Chinese case-control cohort recruited from multiple centers consisting of 364 patients with T1D and 719 unrelated healthy children. We genotyped 55 single nucleotide polymorphisms (SNP) markers located in 16 non-HLA genes (*VTCN1*, *PTPN22*, *CTLA4*, *SUMO4*, *CD274*, *IL2RA*, *INS*, *DHCR7*, *ERBB3*, *VDR*, *CYP27B1*, *CD69*, *CD276*, *PTPN2*, *UBASH3A*, and *IL2RB*) using SNaPshot multiple single-base extension methods. After multivariate analysis and correction for multiple comparisons, we identified the SNP rs2292239 in *ERBB3* gene were significantly associated with T1D. The frequency of the major G allele was significantly decreased in patients with T1D (68.8% in T1D vs 77.3% in controls, OR 0.65, 95% CI 0.53–0.79, P = 0.02), and the minor allele T was associated with an increased risk of T1D (OR 1.55, 95% CI 1.26–1.90, P = 0.02). Our haplotype analysis confirmed that rs2292239 was the primary T1D association locus in our current investigation. These results indicated that the *ERBB3*-rs2292239 was the primary T1D association locus among the investigated 55 SNPs in 16 non-*HLA* genes in Chinese Han population.

## Highlights

• A large scale case-control genetic association study on type 1 diabetes in Chinese investigating on-*HLA* genes.
• rs2292239 in the *ERBB3* gene conferred the primary non-*HLA* association in Chinese type 1 diabetes.
• Markers in the common candidate genes, such as PTPN22, CTLA4, IL2RA, and INS, were not significantly associated with T1D in our Chinese cohort.

## 1 Introduction

Type 1 diabetes (T1D) is an autoimmune disease influenced strongly by genetic factors (Atkinson et al., 2014). It was indicated that the life time concordance rate of T1D related autoimmunity in monozygotic twins can be as high as 65% (Redondo et al., 2008).The incidence of T1D varies remarkably among different ethnic/geographical populations and ranges from around 5/100,000 person-years in East Asian populations to > 40/100,000 person-years in some European populations (Patterson et al., 2009, Lu et al., 2014, Zhao et al., 2014). Although the incidence of T1D is rising worldwide, East Asian populations remained to have low incidence (Lu et al., 2014, Zhao et al., 2014). These facts suggested that the genetic heterogeneity between ethnics can be one of the essential factors that form the incidence gap between populations (Ikegami et al., 2007).

Recent genome-wide association studies (GWAS) revealed many T1D association loci located in genes related to T1D autoimmunity, including *PTPN22*, *CTLA4*, *IL2RA*, *INS*, *CD69*, *ERBB3*, *CYP27B1*, *PTPN2*, *UBASH3A* and *IL2RB* (Barrett et al., 2009, Bradfield et al., 2011, Burren et al., 2011). In addition, there were other studies suggested that small ubiquitin like modifier 4 gene (*SUMO4*) (Guo et al., 2004); members of B7 family, B7-H1 (*CD274*), B7-H3 (*CD276*) and B7-H4 (*VTCN1*) (Dai et al., 2014); genes in vitamin D metabolism and signaling (*DHCR7* and *VDR*) genes (Cooper et al., 2011) were also candidate genes involved in the pathogenesis of T1D.

Chinese Han population is among the East Asians with the lowest incidence of T1D around the world (Lu et al., 2014, Zhao et al., 2014). Similar to other East Asian populations, the Chinese Han maintains at a low level of T1D incidence despite rapid changing in environmental factors (Zhao et al., 2014). In our previous study, we identified among Chinese Han that rs1217419 is the primary T1D association locus in *PTPN22* and revealed the heterogeneity in *PTPN22* association between Chinese Han and Caucasians (Pei et al., 2014). Nevertheless genetic studies using candidate gene approach were likely to create specious results even after statistical adjustment (Qu et al., 2005). Therefore in our current study aiming to minimize the insufficient protection against biased results, we used our multi-center cohort of patients with T1D in the Chinese Han population and multivariate analysis to investigate the genetic association of non-*HLA* genes by genotyping 55 single nucleotide polymorphisms (SNPs) in *VTCN1*, *PTPN22*, *CTLA4*, *SUMO4*, *CD274*, *IL2RA*, *INS*, *DHCR7*, *ERBB3*, *VDR*, *CYP27B1*, *CD69*, *CD276*, *PTPN2*, *UBASH3A* and *IL2RB* genes.

## 2 Subjects and methods

### 2.1 Study design and subjects

We recruited a case-control study cohort involving 364 patients with T1D and 719 unrelated healthy children. These subjects of Chinese Han origin were recruited in six medical centers across China as previously described in details (Pei et al., 2014). The study protocol was reviewed and approved by the medical ethics committee of the Children's Hospital of Fudan University. Written informed consent was obtained from the legal guardian of every participant, or the participants themselves when legally applicable.

### 2.2 Single nucleotide polymorphisms and genotyping

We studied 55 SNPs that are mapped to genes previously implicated in the pathogenesis of T1D (described above in the introduction section and listed in Table 1). These SNPs were retrieved from the HapMap database (HapMap Public Release #27) with correlation coefficient r^2^ > 0.8. We performed genotyping of those SNPs using genomic DNA extracted from 500 μl peripheral blood of the subjects (RelaXGene Blood DNA System, Tiagen, Beijing, China). The genotyping was carried out by SNaPshot multiple single-base extension (SBE) reaction using purified PCR products amplified from genomic regions containing target SNPs as previously described (Pei et al., 2014). Genotype data were derived from the analysis on SBE products by an ABI PRISM 3130XL DNA sequencer (Applied Biosystems) and GeneMapper 4.0 (Applied Biosystems). The genotyping was performed at least in duplicates to avoid ambiguous genotype data.

### 2.3 Statistical analysis

We conducted data analysis using R (version 3.2.3, www.r-project.org). Deviation from the Hardy-Weinberg equilibrium (HWE) was first evaluated using Pearson's χ^2^-test. The allelic association between each SNP and T1D were tested using logistic regression. P values < 0.05 after Bonferroni correction were considered statistical significant. We conducted haplotype analysis using Haploview (version 4.1) with 10,000 permutations; a P-value < 0.05 after Bonferroni correction was considered significant. Odds ratios (OR) and 95% confidence intervals (CI) of each haplotype were calculated using Fisher's exact test by comparing the haplotype frequencies predicted by Haploview.

## 3 Results

### 3.1 Single marker association

We identified three SNPs in *INS* gene that had marginal association with T1D. The frequencies of rs689-T allele, rs1004446-G allele and rs3741208-G allele were higher in T1D as compared to controls (P value 0.01, 0.02 and 0.008 respectively). However these associations were no longer significant after Bonferroni correction (Table 2). In addition, we identified another two SNPs, *PTPN2*-rs2532151 and *UBASH3A*-rs3827233 had weak association with T1D. The frequencies of G allele in *PTPN2*-rs2542151 and G allele in *UBASH3A*-rs3827233 were slightly increased in patients with T1D than in controls but the significance lost after statistical correction for multiple comparisons.

Our results showed that the SNP rs2292239 in *ERBB3* gene were strongly associated with T1D. The G allele in rs2292239 conferred protection against T1D (Table 2). The frequency of the major G allele was significantly decreased in patients with T1D (68.8% in T1D vs 77.3% in controls, OR 0.65, 95% CI 0.53–0.79, Pcorr = 0.02), and the minor allele T was associated with an increased risk of T1D (OR 1.55, 95% CI 1.26–1.90, Pcorr = 0.02).

### 3.2 Haplotype association

To investigate whether there was combinational effect of SNPs that further affected the association between gene and T1D, we conducted haplotype analysis using genotyping data of the above identified four genes (*INS*, *PTPN2*, *UBASH3A* and *ERBB3*). Our results showed that the haplotypes in *INS*, *PTPN2* and *UBASH3A* genes were not associated with T1D (Table 3). However the *ERBB3* haplotype (rs2292239, rs705708 and rs2292238) were significantly associated with T1D. After Bonferroni correction, the G-G-A haplotype were significantly less frequent in patients with T1D than in controls (OR = 0.71; Pcorr = 0.03) and the T-G-A haplotype was positively associated with T1D (OR = 1.53; Pcorr = 0.03).

## 4 Discussion

In this study, we jointly analyzed the association between type 1 diabetes (T1D) and 55 SNPs in 16 non-*HLA* candidate genes. Our results showed that in the Chinese Han population, after using a relative large sized sample and multivariate analysis, the genetic association between non-*HLA* genes and T1D was located primarily in the *ERBB3* gene.

The association between *ERBB3*-rs2292239 and T1D has been identified in large scaled studies in many populations, including Caucasians (allelic OR 1.3) (Barrett et al., 2009, Bradfield et al., 2011, Burren et al., 2011), Japanese (allelic OR 1.5) (Yamashita et al., 2011) and Pakistani (Kiani et al., 2015). Similar to studies in Asian populations, we confirmed that rs2292239 in *ERBB3* was one of the loci with the most significant T1D association among non-*HLA* genes (Yamashita et al., 2011, Kiani et al., 2015). The *ERBB3* gene encodes erb-b2 receptor tyrosine kinase 3 (ErbB3), a member of the epidermal growth factor receptor (EGFR) family (Avraham and Yarden, 2011). Dislike other members in the EGFR family, ErbB3 interacts more specifically with PIK3 regulatory subunits (Jones et al., 2006), which transduce signals downstream to mTOR signaling pathway and in turn regulate insulin production in β-cells and subsequent glucose metabolism (Senniappan et al., 2014). However, the pathogenic role of the intronic *ERBB3*-rs2292239 in T1D is still obscured since it had no apparent effects on *ERBB3* expression in previous systematic genotype-expression studies (Westra et al., 2013, Consortium, G.T., 2015). Although our haplotype analysis revealed that the primary T1D association among the three investigated SNPs in *ERBB3* was located in rs2292239, recent finer mapping of association surrounding *ERBB3* in European population indicated that the association of rs2292239 can be secondary to adjacent SNPs such as rs2271189 and rs11171747 (Keene et al., 2012). Whether these findings apply to East Asian population is still awaiting confirmation from future studies with higher genetic coverage over the *ERBB3* region.

After recent large genetic association studies conducted in Caucasian populations, nearly 60 T1D risk loci have been identified (Barrett et al., 2009, Bradfield et al., 2011, Burren et al., 2011). While these outstanding studies greatly unveiled the underlying mechanism of T1D pathogenesis, replication of their findings in East Asian populations sometimes leads to inconsistent results (Yamashita et al., 2011, Tang et al., 2012, Kiani et al., 2015). In addition, the majority of these susceptible genes have association odds ratios below 1.75 in most case-control studies (Pociot et al., 2010). The genetic effect of each gene alone might be too trivial to ascertain by individual studies. Indeed, while compare to studies scaled similar to ours in the East Asian populations, we were only able to replicate the T1D association in *ERBB3*-rs2292239 however not in *INS* or *IL2A* (Yamashita et al., 2011). Meanwhile, using the current data with expanded genetic coverage, our results revoked the previously reported T1D association in *PTPN22* (Pei et al., 2014). In addition, we did not confirm other common T1D candidate genes such as *CTLA4*, *VDR* and *SUMO4*. Reasons for these discrepancies can be partly subjected to the rarity of minor alleles (such as *INS*-rs689) in the East Asian populations therefore studies results are more likely to be influenced by statistical stringency and other variations across study protocols. Furthermore, the weak association between non-*HLA* susceptible genes made the inter-ethnic association easily influenced by variations of allele distributions across populations (Thomson et al., 2007).

To the best of our knowledge, we have performed the largest genetic association study in Chinese patients with T1D. Although we endeavored to increased protection against biased results, after applying centralizing cohort recourses, multivariate statistical method and a strict statistical adjustment, we believe that the current study is still limited in sample size to reach convincing statistical power to ascertain the association of alleles with lower frequencies. Despite the low incidence of T1D in East Asians creates a difficulty for studies to increase sample size, future investigations in these populations should recruit more subjects and involving larger scale of genetic regions in order to forge the genetic association of T1D in these populations.

In conclusion, the results of our genetic analysis in Chinese Han population showed that the *ERBB3*-rs2292239 was the primary T1D association locus among the investigated 55 SNPs in 16 non-*HLA* genes.

## Funding

This study received financial supported from the Science and Technology Commission of Shanghai Municipality (STCSM 12410709700), Chinese National Twelfth Five Year Key Science and Technology Plan (2012BAI09B00) and National Natural Science Foundation of China (NSFC-81500599).

## Conflicts of interest

The authors declare that there is no conflict of interest related to this article.

## Figures and Tables

**Table 1:** List of studied SNP markers in the candidate genes.

Chromosome	Nearest gene	Marker	MAF in CHB	MAF in CEU
1p13.1	*VTCN1*	rs10158166	0.20 (T)	0.12 (T)
	rs1937956	0.11 (T)	0.30 (T)
1p13.2	*PTPN22*	rs1217385	0.17 (C)	0.44 (A)
	rs2488457	0.33 (C)	0.22 (G)
	rs1217414	0.12 (A)	0.26 (A)
	rs1217419	0.14 (G)	0.48 (T)
	rs3765598	0.20 (T)	0.23 (T)
	rs2476601	0.02 (A)	0.12 (A)
2q33	*CTLA4*	rs5742909	0.11 (T)	0.08 (T)
	rs231775	0.30 (A)	0.39 (G)
	rs3087243	0.20 (A)	0.20 (G)
6q25	*SUMO4*	rs6570965	0.39 (C)	0.36 (A)
	rs237027	0.17 (T)	0.12 (T)
	rs763590	0.10 (T)	0.23 (T)
	rs600739	0.49 (A)	0.08 (G)
	rs237025	0.20 (G)	0.50 (G)
9p24	*CD274*	rs702275	0.29 (G)	0.48 (T)
	rs822338	0.45 (T)	0.23 (C)
	rs2282055	0.39 (T)	0.27 (G)
	rs6415794	0.12 (A)	0.48 (A)
	rs2297137	0.46 (A)	0.22 (A)
10p15	*IL2RA*	rs12251307	0.20 (T)	0.10 (T)
	rs4749955	0.34 (T)	0.44 (C)
	rs3118470	0.45 (T)	0.31 (C)
	rs706778	0.38 (C)	0.40 (T)
	rs2104286	0.11 (C)	0.25 (C)
11p15.5	*INS*	rs689	0.05 (T)	0.24 (T)
	rs7924316	0.46 (T)	0.41 (G)
	rs1004446	0.33 (A)	0.39 (A)
	rs3741208	0.17 (A)	0.36 (A)
11q13.4	*DHCR7*	rs12785878	0.47 (G)	0.27 (G)
12p13	*CD69*	rs3136563	0.47 (G)	NA
	rs4763879	0.48 (A)	0.38 (A)
	rs10844706	0.39 (A)	0.39 (A)
	rs917911	0.35 (T)	0.34 (G)
12q13	*ERBB3*	rs2292239	0.27 (T)	0.33 (T)
	rs705708	0.33 (A)	0.50 (A)
	rs2292238	0.38 (C)	0.41 (C)
12q13.11	*VDR*	rs2228570	0.44 (A)	0.41 (A)
	rs7975232	0.31 (A)	0.43 (C)
	rs1544410	0.04 (T)	0.44 (T)
12q14.1	*CYP27B1*	rs4646536	0.24 (A)	0.34 (G)
15q23	*CD276*	rs11574483	0.11 (A)	0.10 (A)
	rs3825859	0.11 (A)	0.07 (A)
18p11.3	*PTPN2*	rs478582	0.15 (C)	0.46 (C)
	rs2542157	0.26 (G)	0.43 (G)
	rs2542151	0.15 (G)	0.12 (G)
21q22.3	*UBASH3A*	rs2277798	0.40 (G)	0.42 (A)
	rs2277800	0.14 (T)	0.04 (T)
	rs9976767	0.28 (G)	0.40 (G)
	rs3827233	0.33 (C)	0.41 (C)
	rs2839519	0.16 (A)	0.11 (A)
22q13.1	*IL2RB*	rs228942	0.26 (T)	0.16 (T)
	rs228941	0.37 (G)	0.31 (G)

Abbreviations: SNP: single nucleotide polymorphism; T1D: type 1 diabetes; MAF: minor allele frequency; MAF data were derived from Hapmap database (HapMap Public Release #27) and dbSNP build 146; CHB: Han Chinese in Beijing, China; CEU: Utah Residents with Northern and Western European Ancestry.

**Table 2:** Allele and genotype frequencies of SNPs with significant association in patients with T1D and healthy controls.

SNP	Allele/Genotype	T1D (%) n = 364	Controls (%) n = 719	OR (95%CI)	P value	Pcorr
*INS*-rs689	T	98.5	95.2	3.16 (1.65–6.68)	0.01	NS
A	1.5	4.8	0.32 (0.15–0.61)		
TT	97.2	90.5	3.61 (1.82–7.97)	0.002	
TA	2.5	9.5	0.25 (0.11–0.51)	0.0007	
AA	0.3	0.0	NA	NA	
*INS*-rs1004446	G	75.8	71.3	1.26 (1.02–1.56)	0.02	NS
A	24.2	28.7	0.79 (0.64–0.98)		
GG	59.9	50.5	1.47 (1.13–1.92)	0.003	
GA	31.7	41.5	0.65 (0.49–0.86)	0.002	
AA	8.4	8.0	1.06 (0.64–1.71)	0.8	
*INS*-rs3741208	G	84.1	80.2	1.31 (1.03–1.68)	0.008	NS
A	15.9	19.8	0.76 (0.60–0.98)		
GG	70.5	64.3	1.33 (1.00–1.76)	0.05	
GA	27.3	31.8	0.81 (0.60–1.07)	0.1	
AA	2.2	3.9	0.56 (0.22–1.28)	0.2	
*ERBB3*-rs2292239	G	68.8	77.3	0.65 (0.53–0.79)	0.0003	0.02
T	31.2	22.7	1.55 (1.26–1.90)		
GG	47.9	59.8	0.62 (0.48–0.81)	0.0002	
GT	41.7	35.0	1.33 (1.01–1.74)	0.04	
TT	10.4	5.2	2.12 (1.28–3.51)	0.002	
*PTPN2*-rs2542151	T	78.5	83.5	0.72 (0.57–0.91)	0.006	NS
G	21.5	16.5	1.39 (1.10–1.75)		
TT	61.0	69.6	0.68 (0.52–0.90)	0.005	
GT	35.1	27.9	1.40 (1.06–1.85)	0.01	
GG	3.9	2.5	1.58 (0.72–3.41)	0.3	
*UBASH3A*-rs3827233	G	72.0	67.9	1.22 (1.00–1.49)	0.02	NS
C	28.0	32.1	0.82 (0.67–1.00)		
GG	51.0	45.6	1.24 (0.95–1.61)	0.1	
GC	42.1	44.5	0.91 (0.70–1.18)	0.5	
CC	6.9	9.9	0.68 (0.40–1.11)	0.1	

Only significant results are shown. Abbreviations: SNP: single nucleotide polymorphism; T1D: type 1 diabetes; OR: odds ratio; 95% CI: 95% confidence interval; Pcorr: P value after Bonferroni correction (n = 55).

**Table 3:** Haplotype frequencies of *INS* and *ERBB3* in patients with T1D and healthy controls.

Gene	Blocks	Haplotype	Haplotype frequency		OR (95% CI)	P value	Pcorr
			T1D n = 364	Controls n = 719			
*INS*	rs689/rs7924316/rs1004446/rs3741208	ATAG	0.014	0.042	0.32 (0.10–0.84)	0.005	NS
	TTGG	0.558	0.486	1.34 (1.03–1.73)	0.01	NS
*ERBB3*	rs2292239/rs705708/rs2292238	GGA	0.417	0.502	0.71 (0.55–0.92)	0.0006	0.03
	TGA	0.227	0.161	1.53 (1.10–2.13)	0.0006	0.03

Only significant results are shown. Abbreviations: T1D: type 1 diabetes; OR: odds ratio; 95% CI: 95% confidence interval; Pcorr: P value after Bonferroni correction (n = 55).
